# “Visual thinking strategies” improves radiographic observational skills but not chart interpretation in third and fourth year veterinary students

**DOI:** 10.3389/fvets.2024.1480301

**Published:** 2024-12-09

**Authors:** Jacob Wolf, Michelle Tillander, Katharine Peper, Victoria Phillips Kastenholz, Vivian Lantow, Charlie Classe, Yihan Jiang, Elayne Colon

**Affiliations:** ^1^College of Veterinary Medicine, University of Florida, Gainesville, FL, United States; ^2^School of Art and Art History, College of the Arts, University of Florida, Gainesville, TX, United States; ^3^College of Education, University of Florida, Gainesville, FL, United States

**Keywords:** art, radiograph (X-ray), visual thinking strategies, clinical judgment, visual literacy and critical analysis, emergency and critical care

## Abstract

The ability to observe and interpret images and clinical information is essential for veterinarians in clinical practice. The purpose of this study is to determine the utility of a novel teaching method in veterinary medicine, the incorporation of art interpretation using the Visual Thinking Strategies (VTS), on students’ observational and clinical interpretation skills when evaluating radiographs and patient charts. Students were asked to observe and interpret a set of radiographs and a patient chart, subsequently involved in art interpretation using VTS, and then asked to observe and interpret a different set of radiographs and a different patient chart. Qualitative and quantitative analysis was performed, including scoring of observations and interpretations by a radiologist and emergency and critical care resident. For radiographs, observation and interpretation scores increased significantly after VTS. There was no change in patient chart observation or interpretation scores after VTS. Broadly, VTS provided creative thinking and visual literacy exercises that students felt pushed students them to think more openly, notice subtleties, use evidential reasoning, identify thinking processes, and integrate details into a narrative. However, its impact on clinical reasoning, as assessed by chart observation and interpretation scores, was uncertain. Further studies are needed to determine the optimal way to incorporate art interpretation in the veterinary medical curriculum.

## Introduction

Veterinarians employ observational and interpretative skills in various contexts. Every day, veterinarians may be asked to interpret a multitude of images, including fundic exams, electrocardiogram strips, diagnostic imaging modalities (radiographs, ultrasound, computed tomography), patient charts, and cytology. Veterinary radiologists record a list of observations and then, from those observations, build a clinical interpretation for the clinician to use. Similarly, veterinarians may examine a patient chart, codify a list of observations or abnormalities, and then develop a clinical interpretation (problem list, differential diagnoses, summary statement, etc.) using clinical reasoning.

Additional methods to improve observational and clinical reasoning skills among veterinary students are needed. Teaching clinical reasoning is complex and challenging due to myriad factors, including the difficulty of explaining cognitive processing, ambiguity, and lack of instructor training (1). The inclusion of the arts and humanities in medical education is proposed to improve observational skills and promote clinical reasoning in learners (2, 3). It is proposed that the arts and humanities may augment clinical reasoning by both enhancing a learner’s “ways of knowing” and by focusing inquiry on the particular, rather than the general (2).

Art interpretation has been extensively studied in medical education as a method to increase observational and interpretative skills, particularly in visual fields such as radiology, dermatology, and ophthalmology. Many of these studies have shown improvements in participant visual analysis with a particular emphasis on improved observational skills in participants (2, 4–7). To date, there has been little research into the incorporation of art into a veterinary curriculum and no research into the inclusion of VTS specifically into a veterinary curriculum (8–10). Visual Thinking Strategies (VTS) is a form of art interpretation that was developed by museum educator Philip Yenawine and psychologist Abigail Housen (11). The VTS approach builds capacity to stay focused, breaking the habit of quickly grasping something, and connecting ideas—it prompts the learners to notice what is happening, not push a solution. With VTS, a facilitator directs participants to consider three carefully constructed, open-ended questions:

*What is going on in this picture?* The initial question aims to open the discussion among participants and invite a variety of comments, ranging from colors, contextual information, to shapes and feelings. It is meant to probe directly for meaning rather than prompt a list of observations.

*What do you see that makes you say that?* A follow-up question is meant to encourage participants to provide visual evidence to strengthen their interpretations.

*What more can you find?* The final question has the goal of encouraging the discussion to become more well-rounded and intuitive (11). In various populations, including undergraduate education, medical education, nursing, and medical residencies, VTS has been shown to increase creativity, observational skills, critical thinking, problem solving, listening and communication, and promotes an openness toward other viewpoints (4, 12).

Numerous studies in human medicine have identified benefits of VTS on medical students, nursing students, pharmacy students, and dermatology residents, including more in-depth analysis, improved critical thinking, and improved observational or visual diagnostic skills (4, 13–16). In one study, medical students increased the amount of time they spent evaluating patient images, the number of words used to describe the images, and the number of observations about those images after art interpretation with museum facilitators (17). A similar study using VTS specifically found that not only did the number of observations increase, but their ability to describe what was happening and where it was located improved following VTS (18). Likewise, a study of first-year medical students found that VTS increased the number of words to describe radiographs, the time spent evaluating the radiographs, and the number of clinically impactful observations (19). A study evaluating medical students found that visual art interpretation decreased use of subjective terminology when describing patients, increased comprehensive interpretations of the patient (rather than focused exclusively on the medical condition), increased speculative thinking to try to form a differential list, and incorporated more visual analogies (5). A preliminary study of VTS with internal medicine residents found that the learners felt they had to confront ambiguity and synthesize multiple meanings in the paintings. These are challenges that are confronted each day with patient care (20).

This study examined the impact of VTS on veterinary students’ observational and interpretative skills as applied to thoracic radiographs and patient charts in a veterinary curriculum. There are limited studies in veterinary medicine that have evaluated the influence of art interpretation on the ability of students to observe abnormalities on images, such as radiographs or a patient chart, and synthesize that into an understanding of the patient disease process. We hypothesized that inclusion of VTS in rounds sessions for third- and fourth-year veterinary students would lead to improved observational and interpretative skills for both radiographic and chart interpretation.

## Methods

This study received an exemption from the University of Florida Institutional Review Board based on the research criteria. Students were included in the study during their 2-week emergency and critical care rotation. Verbal informed consent was obtained upon inclusion. The study was conducted by a veterinary criticalist and a facilitator from the College of the Arts and Art Education (CAAE) on third-and fourth-year veterinary students. The study was conducted during their normal rounds time on the rotation, and groups typically included 3–5 students at a time. Veterinary students had already completed their pre-clinical courses in diagnostic imaging and were trained in the Subjective, Objective, Assessment, and Plan (SOAP) format prior to enrollment. Participation was voluntary; students could decline to be included in the research component of rounds. All data collection was de-identified.

Students were administered a written pre-survey (Supplementary material S1), which included demographic questions and prompted them to reflect upon their experiences with the arts. Students were then shown a two-view set of thoracic radiographs (right lateral and ventrodorsal view) on a screen and were asked to list their observations (i.e., bronchial pattern, interstitial pattern, alveolar pattern, pattern distribution, etc.) and clinical interpretation or diagnosis based on the set of radiographs. Students were next show a patient chart and were asked to list their observations and clinical interpretation of the patient’s condition. The chart contained vital parameters, fluid rates, non-invasive blood pressure results, and patient information (vomiting, diarrhea, urination habits, etc.) over a 24-h period. For both the radiographs and the chart, only a brief history was provided. The radiographs depicted a dog with eosinophilic bronchopneumopathy, and the patient chart depicted a dog that entered hypovolemic shock during hospitalization for parvovirus. Students were provided 3 min each to view and write down their observations for the radiographs and patient chart.

**Figure 1:**
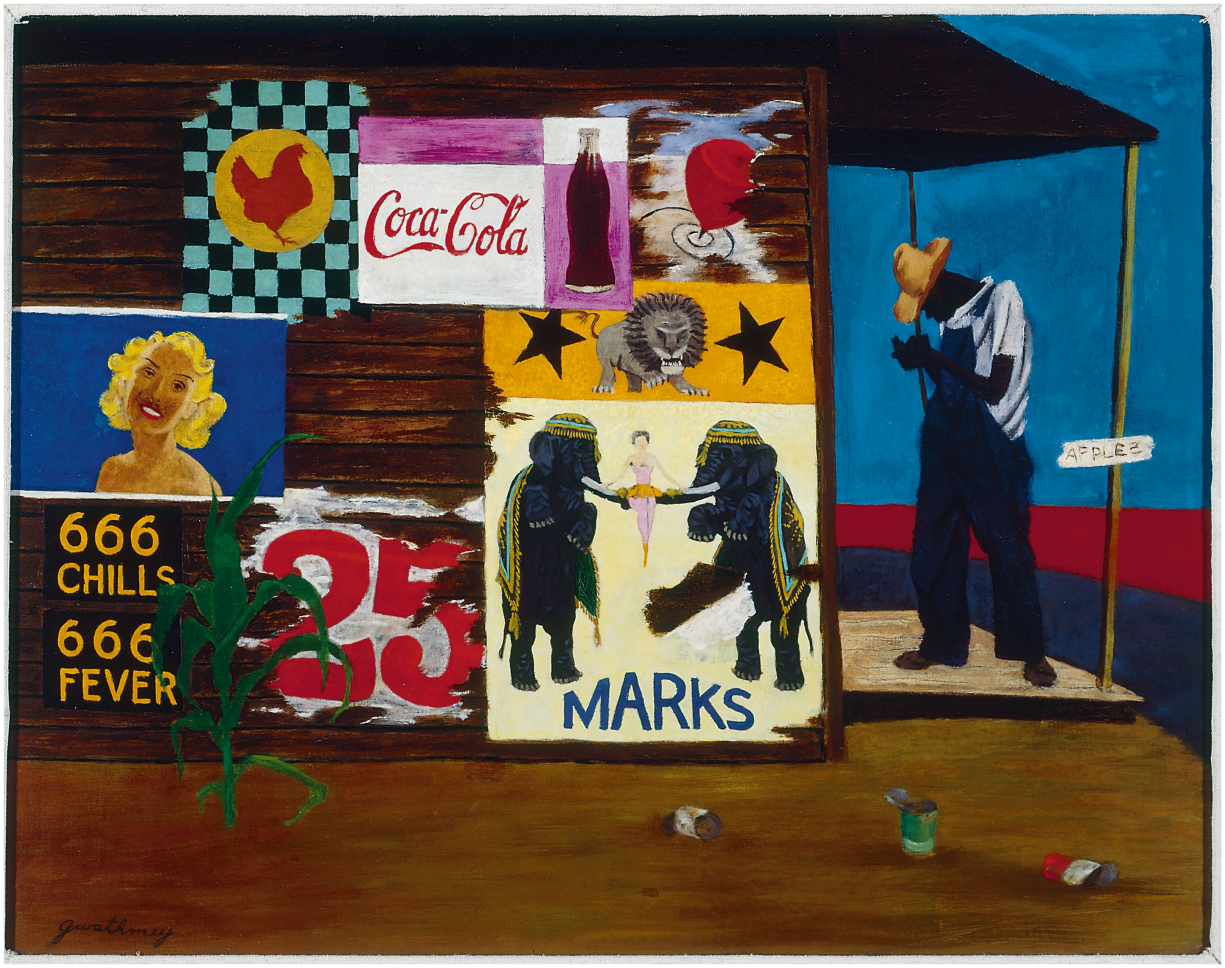
© 2024 Estate of Robert Gwathmey/Licensed by VAGA at Artists Rights Society (ARS), NY. Reproduced with permission.

Following this, the facilitator from the CAAE conducted VTS using the oil painting *Marketing* by Robert Gwathmey (Figure 1), which was projected. The social realist painting was selected because the composition provided accessible visual content for everyone, sustained human interest, the imagery was open to interpretation though a variety of valid readings, and offered the possibility of several levels of meanings. Students were asked to write their own thoughts about the image on an index card over a 3-min period. The group was then invited to discuss the painting together for approximately 20–30 min. During this section, the facilitator repeated the three VTS questions described in the Introduction, provided positive affirmation for student contributions, and paraphrased the student responses back to the group. The questions were repeated numerous times and directed at various students to prompt involvement from each student and to encourage the students to dig deeper into the image. The facilitator maintained neutrality but showed interest in each comment and pointed at the image repeatedly during the comments to maintain the group’s focus on the artwork. Once the students had no more comments, they were asked again to write their thoughts about the artwork on the back of the original index card; the index cards were then collected.

Lastly, students were administered a post-survey (Supplementary material S2), which included questions about their experience with VTS. The same activity from before VTS was then repeated with a different set of thoracic radiographs and a different patient chart. Only a brief history was provided for each patient. The radiographs depicted a dog with left-sided congestive heart failure secondary to mitral valve disease and the patient chart depicted a dog that developed volume overload and oliguric renal failure during hospitalization for acute kidney injury. The second set of a patient chart and radiographs were considered similar difficulty to the first patient chart and radiographs. Students were provided 3 min each to write down their observations for the radiographs and patient chart.

All responses were recorded. Students’ radiographic observation and clinical interpretation scores were graded based on the rubric created by the American College of Veterinary Radiology Certifying Examination Examiner Scoring Guidelines (Supplementary material S3). A modified version of this was used to score the observation and clinical interpretation of the patient charts. A radiologist (KP) and emergency and critical care resident (VP) scored each response. The evaluators were blinded to the order in which the patient chart and radiograph were evaluated.

### Data analysis

Responses were transferred from the written forms to Excel. Grounded theory was used to analyze pre-and post-survey responses iteratively to identify patterns and concepts and look for similarities, differences, and core categories (21). Once the data were not providing any new insights, we had reached theoretical saturation for this set of data and stopped analyzing data. For this research, Charmaz’s approach to grounded theory aims for theory construction, not description, and encourages a holistic understanding of phenomena by integrating multiple perspectives and recognizing that knowledge is not fixed, but rather shaped by ongoing interactions within specific contexts. The use of grounded theory, particularly through a constructivist perspective facilitates the analysis and interpretation of our survey data and discussions by emphasizing the co-construction of knowledge by participants, acknowledging the dynamic and context-dependent nature of reality. By coding data and identifying themes, as researchers we are able to construct explanatory models that capture the multifaceted experiences and perceptions of study participants in how they come to know, enabling a richer understanding of phenomena and the complex interplay of meanings derived from lived experiences and the analysis of art work with VTS and finally the diagnosis of the small animal radiographs (22). Pre-and post-test scores on clinical interpretation and observation were analyzed using paired samples t-tests with a web-based data analysis software program.^1^ Their score changes were tested for statistical significance using paired *t*-tests at a threshold *p*-value <0.05. Additionally, 5-point Likert scale questionnaires gathered student self-perceptions of the VTS training. Responses options consisting of “Strongly disagree,” “Somewhat disagree,” “Neither agree nor disagree,” “Somewhat agree,” and “Strongly agree” were assigned numeric scores from 1 to 5, respectively.

## Results

### Demographic information and quantitative results

A total of 37 students were included in the study. The mean age was 26 years old (range: 23–35 years); 8 students (22%) identified as male, and 29 students (78%) identified as female. When discussing experience with the arts, 4 students (11%) had formal training in the arts (undergraduate or graduate classes) and 15 students (41%) had informal experience in the arts. Seven students (19%) previously participated in art interpretation.

For radiographic observations (*n* = 35, due to two students’ incomplete answers), mean scores increased from 1.23 before VTS to 2.26 after VTS (*p* < 0.001). For radiographic interpretation (*n* = 35, due to two students’ incomplete answers), scores increased from 0.14 before VTS to 0.74 after VTS (*p* < 0.01). No significant difference (*p* = 0.554) was noted for patient chart observations (*n* = 36, due to one student’s incomplete answers) before VTS (1.92) and after VTS (2.03). No significant difference (*p* = 0.314) was noted for patient chart interpretation (*n* = 36, due to one student’s incomplete answers) before VTS (1.42) and after VTS (1.61). See Table 1 for complete statistical information.

**Table 1:** Statistical analysis of students pre and post intervention in radiographic observations and interpretations and patient chart observations and interpretations.

Theme	Time point	Mean	Standard deviation	*p*-value
Radiographic observations (*n* = 35)	Pre-intervention	1.23	1.09	<0.001*
	Post intervention	2.26	1.15	
Radiographic interpretation (*n* = 35)	Pre-intervention	0.14	0.36	0.006*
	Post intervention	0.74	1.12	
Patient chart observations (*n* = 36)	Pre-intervention	1.92	0.94	0.554
	Post intervention	2.03	0.97	
Patient chart interpretations (*n* = 36)	Pre-intervention	1.42	0.87	0.314
	Post intervention	1.61	0.84	

* indicates values that achieved statistical significance.

Word counts were evaluated as a proxy for engagement. The average word count for radiographic observations and interpretation increased after VTS from 13 words/student to 23 words per student. The average word count for chart observation and interpretation remained static before and after VTS at 29 per student. The average number of words used to describe the painting increased from 34 words/student to 124 words per student.

In the post-survey, 26 students (70%) responded that they felt VTS changed their approach to the clinical interpretation and observation of the radiographs and patient charts, eight students (22%) did not think that it changed their approach, and three students (8%) were unsure. Thirty students (81%) either agreed or strongly agreed that they found VTS fun; only two students (5%) disagreed that they found VTS fun. Twenty-six students (70%) either disagreed or strongly disagreed that VTS was stressful; six students (17%) agreed or strongly agreed that VTS was stressful. Twenty students (54%) agreed or strongly agreed that VTS was helpful, 13 students (35%) were unsure, and 3 students disagreed (8%) that VTS was helpful.

### Qualitative results

When using grounded theory to analyze the qualitative narrative data, the categories that emerged from this data were that most students felt that VTS encouraged them to think more openly, imaginatively, and in an integrated manner. The VTS process pushed students outside their usual thought patterns. Self-described visual learners especially appreciated the visual components of VTS. Critical thinking skills like puzzle solving, integrating details, and dynamic thinking were seen as enhanced by VTS; however students responded that they would require repetition and practice to increase utility. Students overwhelmingly valued consulting peers during VTS exercises to gain other perspectives. Many students believed VTS made them pay closer attention to radiographic details and subtle findings that aided in their evaluation. Grounded theory allowed us to unravel the immediate impact research participants had with thinking through interpretations and experiences using VTS. There were mixed opinions in the data on whether VTS significantly impacted clinical interpretation and observations. This is to be expected as the research would need to be conducted long term, following the participants continued clinical experiences and observations.

When evaluating the initial art interpretation on the index cards, some students listed straightforward descriptive elements devoid of a sentiment or questions around broader significance. For example, “*south or Midwest, corn, not present day, side of a general store.”* This statement lacks movement past factual contents and into purpose-driven, interpretive space. While accurately naming components across artistic dimensions is fundamental, failing to then synthesize details into potential cultural narratives or symbolic messages demonstrated limited complexity.

Conversely, other student responses exhibited an effort to interweave selected details into subjective narrative or speculate more abstractly beyond surface content, providing a lens into higher-order observation and meaning-making skills. For example, “*It makes me think of an old southern roadside stand. It looks quiet but a little run down, like no one passes by much anymore. The artist took time to show the wear on the posters and the trash on the ground.*” The attention to mood-capturing descriptors and hypothesizing the artist’s reflects evaluation occurring beyond basic identification. Similarly, a student response that stated “*Man maybe at a store or produce stand…The 666 chills and fevers sign is funky in a weird way. I cannot tell what the person is doing, maybe counting money*” notably moves beyond dry description and into brainstorming esthetic intentions. Creatively hypothesizing on ambiguous details displays an invitation for uncertainty, a catalyst for unearthing richer insights across analytical domains. These qualities distinguished students who approached the art with more sophisticated analysis.

## Discussion

This study builds upon previous studies in veterinary medicine to demonstrate through quantitative and qualitative methods that art interpretation can improve radiographic observations and interpretations for veterinary students. Students felt that VTS enhanced their attention to subtle findings on radiographs and helped them evaluate the entire “picture.” Students also overwhelmingly found the activity fun, non-stressful, and felt that it was helpful for their approach to radiographs and chart interpretation. These findings should prompt discussions about the inclusion of the arts in veterinary curricula.

Through qualitative assessment of comments on the VTS activity, it was identified that visual learners particularly found VTS useful. This warrants further exploration in those who self-identify as visual learners as a method of appealing to and enhancing their learning experience. Furthermore, students reported that VTS prompted puzzle solving, detail integration, communication between peers, and creative thinking. These are all skills that are essential to a clinician and their development can be challenging in a traditional didactic structure. Additional studies to better measure whether improvements in these areas are prompted by VTS are needed.

A previous study found that veterinary students believed they developed enhanced radiographic interpretative abilities after incorporation of the visual arts, though it did not utilize VTS specifically. When matched to a group that did not attend the visual arts seminar, those who attended it had a higher ability to identify abnormalities and better descriptions of the abnormalities on radiographs (8). The improvement in radiographic observation and clinical interpretation skill scores of the current study are consistent with the findings of the previous study. Another study found that fine arts-based observation training and pathology-based observation training improved observational skills in veterinary students enrolled in a cytology course. However, in that study, the fine-arts group had less accurate descriptions than the pathology-based group immediately after the training; this difference was not maintained 4 weeks post-training (10).

The approach that VTS encourages involves “exploration, extraction of relevant information, simplification, and organization of information.” (16, 23) It is postulated that the benefits of VTS may be due to its ability to foster an environment in which the discussions allow time for reconsideration of initial assessments. Also, the facilitator’s paraphrasing may help reduce biases and assumptions made at the outset. These are important qualities to develop in clinicians to avoid bias and pitfalls in diagnostic reasoning (4). Many learners may feel stressed and anxious when asked to interpret radiographs or patient charts. Art interpretation is an area in which most veterinary students are unfamiliar. VTS can help level the playing field between students and minimize the stress and judgment associated with evaluation of an image. This allows students to develop transmissible skills in a less stressful environment (24). Understanding typologies of VTS can help educators tailor their teaching approaches as well as student’s expansion of metacognitive strategies when analyzing artwork and then transferring the topologies to reading radiographs for diagnosis. When focusing on critical thinking and analysis, emphasizing observation and analytical frameworks is key. By fostering personal engagement and self-expression, personal and contextual interpretation strategies become crucial. Students are guided to describe what they see in an artwork without jumping to conclusions. This encourages detail-oriented observation and helps to establish a shared context. After making initial descriptions, students are encouraged to make inferences about the artwork; in responding to “what makes you say this?.” This promotes critical thinking and interpretation, pushing students to connect visual elements with meaning. VTS allows students to relate artworks to their own experiences or emotions. Personal interpretation as a strategy fosters personal connection and makes learning more relevant. Contextual interpretation encourages students to consider the historical, social, or cultural context of an artwork and enhances understanding and appreciation. These typologies combined involve a more holistic strategy when looking and seeing. Critical thinking and analysis, emphasizing observation and analytical frameworks, and personal engagement and self-expression strategies reinforces metacognitive skills and personal growth in understanding, which may be translational skills to other images (i.e., radiographs). Similarly, a study from the Yale University School of Medicine that showed a significant improvement in student observations after art interpretation postulated that the lack of familiarity with artwork by medical students is the beneficial quality. This unfamiliarity prompts the student to evaluate all details of the painting, as they are not biased in thinking which visual components are important or not important (23).

Although this study identified improvement in radiographic observation and clinical interpretation, these findings were not replicated when students evaluated the patient charts. The inclusion of chart interpretation was elected since the patient chart is a visual medium that may have subtle details and a holistic chart interpretation may be representative of clinical reasoning. While students reported that VTS improved their interpretative abilities, this was not borne out in quantitative analysis of the patient chart data. It is possible that a larger study population may have found more impact of VTS on patient chart interpretation, as a slight increase in patient chart interpretation was noted but it did not reach statistical significance. Additionally, the patient chart scores were higher than the radiographic scores. It is possible that students were already much more comfortable with evaluating a patient chart compared to evaluating a radiograph by this point in the clinical curriculum. Repeating this study with pre-clinical veterinary students (first and second year students) may yield different results.

Harvard Medical School has developed an elective course curriculum that directly links VTS with patient evaluation. This novel course adapts the VTS questions to the patient setting to encourage the same thought process as used when interpreting art (25). This may serve as a model for the inclusion of art interpretation in veterinary medical education moving forward. An open dialog between medical educators, including those in veterinary medicine, and educators in the arts and humanities is needed to establish research goals and implement curricular changes. Starting with elective courses that have art and humanities content threaded throughout the four-year veterinary curriculum may be a reasonable starting point.

This study has several limitations. Only one piece of art was used during the study; it is unclear whether interpretation of different or additional artwork would provide the same benefit. However, other studies have used a variety of paintings and have found similar benefits. Additionally, the images and patient chart were not randomized; they were always shown in the same order. Randomization of image and chart order may have reduced bias, as students may have been more comfortable interpreting one image or chart more than the other one. This study did not evaluate the impact of VTS on other visual diagnostics (fundic exam, histopathology, etc.). These areas should be considered for future research endeavors. Finally, this study did not assess long-term radiographic interpretation skills to see if this benefit was retained.

This study found that students reported that VTS provided a creative thinking exercise that pushed them to think more openly, notice subtleties, and integrate details into clinical interpretations. The visual components and peer discussions are valued. A statistically significant improvement in radiographic observations and interpretation was noted in clinical veterinary students; however, this was not replicated in their ability to observe and interpret a patient chart. Overall, VTS was an enjoyable activity that improved radiographic observations and interpretation; its ability to improve clinical reasoning requires further evaluation.

## Data Availability

The raw data supporting the conclusions of this article will be made available by the authors, without undue reservation.

## Footnotes

^1^SPSSAU, QingSi Technology Ltd., Haidian, China.
