# Erratum: “Visual thinking strategies” improves radiographic observational skills but not chart interpretation in third and fourth year veterinary students

**DOI:** 10.3389/fvets.2025.1590961

**Published:** 2025-03-27

**Authors:** Frontiers Production Office

**Affiliations:** Frontiers Media SA, Lausanne, Switzerland

**Keywords:** art, radiograph (X-ray), visual thinking strategies, clinical judgment, visual literacy and critical analysis, emergency and critical care

Due to a production error, the latest and correct version of the Supplementary Material, which includes Supplementary Data Sheets 1, 2, and 3, was not published. These files have now been published, replacing the incorrect Supplementary Material files.

The publisher apologizes for this mistake. The original version of this article has been updated.

