# Supplementary material for: “Visual thinking strategies” improves radiographic observational skills but not chart interpretation in third and fourth year veterinary students
Source: Front Vet Sci. 2024 Dec 9;11:1480301. doi: 10.3389/fvets.2024.1480301 (PMC11664864; doi:10.3389/fvets.2024.1480301)
Supplement: Supplementary file 1 [file Data_Sheet_1.docx]

**Student Survey (Pre)**

Responses are anonymous and your identity is protected. This survey will take approximately 10 minutes.

1. Please give your observations and clinical interpretation of the displayed thoracic radiographs:

1. Please give your observations and clinical interpretation of the displayed patient chart:

1. What year veterinary student are you? _____________________
2. a. Prior to entering UF’s program, how many years of experience did you have in the veterinary field? _______________

b. Describe your prior veterinary experience and employment position(s).

1. What is your age? __________________
2. Please list your sex: __________________
3. Please list your ethnicity: __________________
4. Prior to entering veterinary school, did you have any formal training in the humanities or visual arts? Yes No
5. If so, list degree(s) and major(s)/minor(s):

11. Do you have any informal experience or interest in the visual/performing arts? Yes No

a. If so, please describe:

1. a. Number of languages spoken: _______________

b. Other than English, please list: __________________

1. Have you previously participated in the interpretation of art? Yes No
2. Please describe:
