# Supplementary material for: “Visual thinking strategies” improves radiographic observational skills but not chart interpretation in third and fourth year veterinary students
Source: Front Vet Sci. 2024 Dec 9;11:1480301. doi: 10.3389/fvets.2024.1480301 (PMC11664864; doi:10.3389/fvets.2024.1480301)
Supplement: Supplementary file 2 [file Data_Sheet_2.docx]

**Student Survey (Post)**

Responses are anonymous and your identity is protected. This survey will take approximately 15 minutes.

1. Please give your observations clinical interpretation of the displayed thoracic radiographs:
2. Please give your observations and clinical interpretation of the displayed patient chart:
3. Do you believe Visual Thinking Strategies (VTS) changed your approach to critical thinking, clinical interpretation, or observation process? If so, please describe how.
4. Please rate the following:

| **I found VTS fun** | Strongly disagree | Disagree | Neither agree nor disagree | Agree | Strongly agree |
| --- | --- | --- | --- | --- | --- |
| **I found VTS stressful** | Strongly disagree | Disagree | Neither agree nor disagree | Agree | Strongly agree |
| **I found VTS helpful to my clinical practice** | Strongly disagree | Disagree | Neither agree nor disagree | Agree | Strongly agree |

1. Any other comments?
