# Supplementary material for: “Visual thinking strategies” improves radiographic observational skills but not chart interpretation in third and fourth year veterinary students
Source: Front Vet Sci. 2024 Dec 9;11:1480301. doi: 10.3389/fvets.2024.1480301 (PMC11664864; doi:10.3389/fvets.2024.1480301)
Supplement: Supplementary file 3 [file Data_Sheet_3.pdf]

ACVR Certifying Examination
Examiner Scoring Guidelines

Observation (weighting – 40%)

Points	*Guideline*
0	Total failure (did not identify any abnormalities); normal findings reported as abnormal; or did not review case.
1	Missed or incorrect findings have a detrimental impact on case. Candidate identifies only minor abnormalities.
2	Missed or incorrect findings have moderate impact on case. Candidate identifies some major or minor abnormalities.
3	Missed or incorrect findings have minimal to no impact on case. Candidate identifies most major and some minor abnormalities.
4	Candidate accurately describes all major and most minor abnormalities.

Synthesis (weighting – 60%)

Points	*Guideline*
0	Total failure. Pathological significance of major abnormalities are not recognized or are interpreted incorrectly
1	Pathological significance of some major abnormalities incompletely recognized; incomplete or some incorrect differential diagnoses
2	Pathological significance of most major abnormalities recognized; plausable differential diagnoses but incorrect prioritization
3	Pathological significance of all major abnormalities recognized; accurate prioritized differential diagnoses
